# Epidemiological investigation of two parallel gastroenteritis outbreaks in school settings

**DOI:** 10.1186/1471-2458-13-241

**Published:** 2013-03-19

**Authors:** Kassiani Mellou, Theologia Sideroglou, Maria Potamiti-Komi, Petros Kokkinos, Panos Ziros, Theano Georgakopoulou, Apostolos Vantarakis

**Affiliations:** 1 Environmental Microbiology Unit, Department of Public Health, School of Medicine, University of Patras, Patras, Greece; 2 Hellenic Center for Disease Control and Prevention, Athens, Greece

**Keywords:** Community outbreaks, Gastrointestinal infections, Waterborne viruses, Norovirus, School settings

## Abstract

**Background:**

Two parallel gastroenteritis outbreaks occurred in an elementary school and a neighboring kindergarten in Kilkis, Northern Greece in 2012. The aim of the study was the investigation of these two parallel outbreaks as well as their possible source.

**Methods:**

Two retrospective cohort studies were performed to identify the mode and the vehicle of transmission as well as the possible connection between them.

**Results:**

Elementary school and kindergarten populations of 79.9% (119/149) and 51.1% (23/45) respectively, participated in the study. Case definition was satisfied by 65 pupils from the elementary school and 14 from the kindergarten. For elementary school, 53 cases were considered primary cases of the outbreak and were included in the analysis. Based on the results of the multivariate analysis, consumption of tap water was the only statistically significant independent risk factor of gastroenteritis (RR = 2.34, 95% C.I.: 1.55-3.53).; a finding supported by the shape of the epidemic curve which referred to a common point source outbreak with secondary cases. For kindergarten, no statistically significant risk factor was identified, and the epidemic curve supported a person-to-person transmission according univariate analysis. Norovirus GI and GII and human Adenovirus were detected by Real Time PCR in stool samples from seven children of elementary school, but stool samples were not collected by children of the kindergarten.

**Conclusions:**

Even though the etiological agent of the outbreak was not verified, combined epidemiological and laboratory results were in favor of a waterborne viral gastroenteritis outbreak at the elementary school, followed by a person to person spread at the kindergarten.

## Background

Viral gastroenteritis is a major health problem worldwide. Viruses cause both seasonal acute gastroenteritis and occasional outbreaks associated with the consumption of contaminated food or water [1]. These outbreaks involve a number of high-risk groups, particularly young children, the elderly and immunocompromised patients [2-6]; they are frequent in semi-enclosed environments, such as school settings due to the increased number of people and the increased personal contacts among them. However, there is not a clear picture on the aetiological agents and source of infection in several outbreaks occurring in school settings because of underreporting and incomplete epidemiological and laboratory investigation of the reported ones. Regarding the source, a few publications refer to waterborne viral outbreaks in such settings [7-9].

In Greece, viral gastroenteritis outbreaks are generally under-reported [10-13] even though clusters of non-bacterial gastroenteritis cases are included in the mandatory notification system of the Hellenic Center for Disease Control and Prevention (HCDCP). To our knowledge no epidemiological studies have been published referring to viral outbreaks regarding a Greek school setting.

On 16 January 2012, HCDCP was informed on an increased number of pupils with gastroenteritis at an elementary school of Nea Santa Kilkis, a village with 1,538 residents, in Northern Greece. The reported symptoms were mild (mainly vomiting and diarrhoea), and supportive of a viral origin. Recommended pre-investigation interventions included the adoption of strict personal hygiene standards, disinfection of shared environmental surfaces, and exclusion of ill persons from school during their illness and for at least 48 hours after resolution of symptoms. A neighboring to the elementary school kindergarten was also contacted to ask for gastroenteritis cases in pupils or staff members and cases of gastroenteritis were confirmed.

The Public Health Department (PHD) of Kilkis informed HCDCP that there had been an increased occurrence of viral gastroenteritis after New Year’s Eve in the community but most of the cases had not visited a medical service. This increase was considered to be inside the limits of the expected for this time of year increase of viral gastroenteritis.

According to the results of the initial investigation of the outbreak at the elementary school, there were no shared meals and children were allowed to consume only the food brought by their home. Cases were almost simultaneously reported by the eight classes of the school and children of different classes did not have any common school activities the days that preceded the occurrence of the first cases. Furthermore, the cases occurred after the school was closed for 15 days for Christmas and New Year’s Eve holidays with the first cases experiencing symptoms late at night of the first day back to school. Initial investigation was against the hypothesis that a pupil or teacher could have been the source of the outbreak since no teacher was included among the first cases based on the reported onset date of symptoms, and the first children that developed symptoms were from different classes, were not family members or friends and had not participated to a common activity. Local public health authorities and teachers suspected that the heavy snowfall and the low temperature may have caused problems to the public water supply system that became obvious at the school as the tap water was not used for two weeks.

Further epidemiological investigation of the outbreak was decided as: a) attack rate of gastroenteritis was high in the elementary school and many cases occurred almost simultaneously, b) the source of the outbreak was not clear, c) outbreaks like this are rarely investigated in Greece and little information can be found in the literature, and d) cases also occurred in the neighboring to the school kindergarten. The objectives of the investigation were to identify the mode and the vehicle of transmission in both settings and also the possible connection between them.

## Methods

### Epidemiological investigation

Listing of all cases and of basic information (pupil/student, onset date of symptoms, class) was requested and new cases were daily recorded and reported to the HCDCP via a structured form.

Two retrospective cohort studies were conducted, one at the elementary school and one at the kindergarten. Gastroenteritis cases were recorded from the beginning of January to the end of January 2012.

A case of gastrointestinal illness for both studies was defined any pupil or staff member who reported vomiting and/or diarrhoea between 09 and 24 January 2012.

For the elementary school, a primary case was defined as any pupil or staff member who experienced gastroenteritis symptoms (vomiting and/or diarrhoea) between 09 and 16 January 2012, and secondary case as any pupil or staff member who experienced symptoms after the 16^th^ of January.

A standardized questionnaire was developed for data collection. It was stressed that the mothers (or guardians) of the children had to complete the questionnaires because of their young age. Questions focused on: 1) demographic data (sex, age), school grade, classroom, 2) illness onset, symptoms, symptoms’ duration, hospitalization, 3) potential risk factors (water consumption, school activities, food consumption, ill contacts). Ill contacts (cases) were defined as persons at school, in the family or among friends with diarrhoea or vomiting. Participants were asked to specify if the contact was taken place inside or outside the school setting. All participants were informed regarding the purpose of the study and consent was asked in order to participate.

### Statistical analysis

Epi Info 3.5.3 was used for data entry and STATA v11.0 (Stata Corporation, Texas, USA) for statistical analysis. Attack rates (AR) per risk factor were calculated. Univariate analysis was performed, calculating relative risks (RR) and 95% confidence intervals (CI) for dichotomous individual exposures. In addition, logistic univariate analysis was used to calculate point estimates including their 95% CI for discrete variables. For the elementary school univariate analysis was performed separately for primary and secondary cases. Multivariate analysis was performed, including the variables that were found to be significant in univariate analysis. Variables statistically significant at p ≤ 0.05 were included in the multivariable model. In the latter model, an association was considered statistically significant when p ≤ 0.05. The multivariable analysis was performed by multiple logistic regressions using backwards elimination.

### Environmental investigation

Data regarding the schools water supply system and possible problems during the previous month of the increase of the cases were recorded. The DPH was asked whether there had been any recent testing of the potable water. Meteorological data were also collected for the same period. On January 17, the DPH conducted a sanitary inspection of the schools, and also performed water sampling. Two tap water samples (10 L each) were collected from the elementary school and the kindergarten on 17 January and were sent to laboratory into cool boxes. An additional water sampling was performed in the local village and also subjected to standard bacteriological analysis (E.coli, Enterococci and Salmonella) according to guidelines [14].

### Laboratory investigation

The skimmed milk (SM) flocculation procedure was used for the detection of viruses in water samples [15]. Viral molecular detection was based on TaqMan® assays previously described by Hernroth et al. (2002) for hAdV [16], Svraka et al. (2007) for NoV GI [17], and da Silva et al. (2007) for NoV GII [18]. The analysis method is accredited by ISO 17025.

Faecal samples were tested for *Salmonella* spp. according to standard procedures and the selected viral targets (hAdVs, NoVs), as described above.

### Ethical statement

According to the Greek legislation, (HCDCP) is the competent authority for outbreak investigation. The school board and the parents' committee gave us their permission to investigate the outbreak via a structured questionnaire and no further approval was requested. In order to reassure informed consent, the questionnaire was accompanied with a letter for parents explaining the scope of the study, clearly stating that they had the choice of not participating to it. Finally, the study was performed in compliance with the Helsinki Declaration; collected data were anonymous and were used only for the purposes of the outbreak investigation.

## Results

### Epidemiological investigation

#### Elementary school

The elementary school population comprised of 149 people, 130 of which were pupils, 18 members of the teaching staff and one was a cleaning staff member.

**Figure 1:**
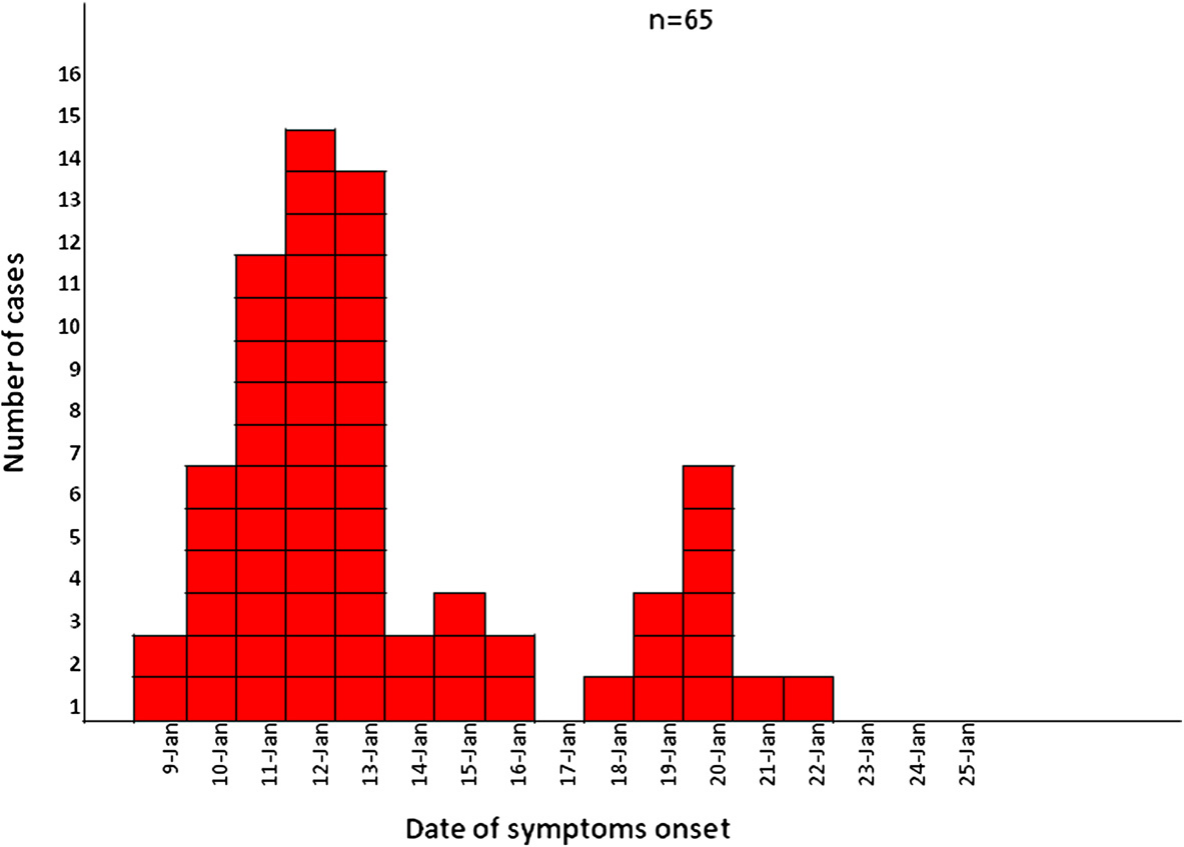
Date of illness onset for gastroenteritis cases (n = 66), Elementary School, Nea Santa, Kilkis, January 2012.

The total response rate was 79.9%. Information was acquired for 103 pupils (response rate: 79.2%), 14 teachers (response rate: 77.8%) and one staff member (100%). Five people were excluded from the study because they might have been community cases based on their history of ill contacts and one because it did not meet the case definition criteria. The median age of the 113 participants of the study was 10 years (range: 6–51 years) and 97 (85.8%) of them were less than 12 years old; 72 (63.7%) were females. A total of 65 persons were found to meet the case definition (attack rate: 57.5%). Distribution of cases by date of symptoms’ onset is presented in Figure 1. Figure 1 shows that the peak of the outbreak was on 12/01/2012. The shape of the epidemic curve is compatible with a common point source outbreak (53 primary cases) followed by a secondary person-to-person transmission (12 secondary cases). The attack rates among males and females were 73.2% and 48.6%, respectively. The vast majority of the reported cases (95.4%) were pupils; and the total attack rate for the student population was 63.9%. Of the 14 members of the teaching staff only one reported symptoms (attack rate: 7.1%), as well as the one member of the cleaning staff. Cases were reported from all classes. Attack rates by class ranged from 50% to 77%. The temporal distribution of cases by class did not depict any specific transmission pattern from one class to another. The main reported symptoms of cases are presented in Table 1. The mean number of vomiting per day was 3 (range: 1–20), while the mean number of diarrhoeas per day was 3 (range: 1–25). The duration of illness ranged from 1 to 12 days, with a median of 2 days. Only one case (1.6%) was hospitalized. Sixty two cases (95.4%) remained at home for recovery, while the median duration of absence from school was 4 days (range: 1–14). Twenty eight cases (43.1%) reported they had a person in their environment with gastroenteritis symptoms before the onset of their symptoms, while forty five cases (70.3%) reported a person in their environment with gastroenteritis after the onset of their symptoms. The univariate analysis that was conducted for primary cases revealed as statistically significant factors both the consumption of water from the tap of the elementary school (RR = 2.09, 95% C.I.: 1.52-2.87), and the consumption of bottled water (RR = 0.63, C.I.: 0.48-0.84) (protective factor). Attack rates and relative risks are summarized in Table 2.

**Table 1:** Reported clinical symptoms of gastroenteritis cases of the Elementary School (n = 65) and the Kindergarten (n = 14), Nea Santa, Kilkis, January 2012

**Symptom**	**Number of cases (%)**	
	**Elementary School**	**Kindergarten**
Vomiting	51 (77.3)	11 (78.6)
Diarrhea	49 (74.2)	7 (50.0)
Abdominal pain	35 (53.0)	9 (64.3)
Weakness/Malaise	35 (53.0)	10 (71.4)
Nausea	28 (42.4)	5 (35.7)
Fever (>38,0°C)	20 (30.3)	5 (35.7)
Arthralgia	13 (19.7)	4 (28.6)

**Table 2:** Results of the univariate analysis (n = 53), Elementary School, January 2012

	**Exposed**			**Non exposed**				
***Risk factors***	***Cases***	***Total***	AR*%	***Cases***	***Total***	AR*%	***RR****†*	***95% CI‡***
**Consumption of tap water from the school**	32	45	71.1	20	66	30.3	**2.35**	1.56-3.54
**Consumption of bottled water**	27	77	35.1	25	35	71.4	**0.49**	0.34-0.71
Consumption of water from the cooler in the teaching staff room	5	11	45.5	47	100	47.0	0.97	0.49-1.91
Another person at approximate environment with gastroenteritis symptoms before the onset of symptoms	25	31	80.7	28	42	66.7	1.21	0.92-1.59
Participation in tutorial lessons (after school)	16	33	48.5	36	73	49.3	0.98	0.64-1.50
Participation in athletic activities	11	23	47.8	41	83	49.4	0.97	0.60-1.56
Visit to playground	1	2	50.0	51	104	49.04	1.02	0.25-4.13

*Attack Rate *†Relative Risk ‡ 95% Confidence Interval*

In the final model of the multiple logistic regression analysis, consumption of tap water, consumption of bottled water (protective factor) and contact with a gastroenteritis case prior to disease onset were included. Based on the results of the multivariate analysis consumption of tap water was the only statistically significant independent risk factor of gastroenteritis (RR = 2.34, 95% C.I.: 1.55-3.53).

All secondary cases reported close contact with a primary case (ill classmate) the two days before their disease’s occurrence. None reported contact with ill persons outside the school environment.

#### Kindergarten

**Figure 2:**
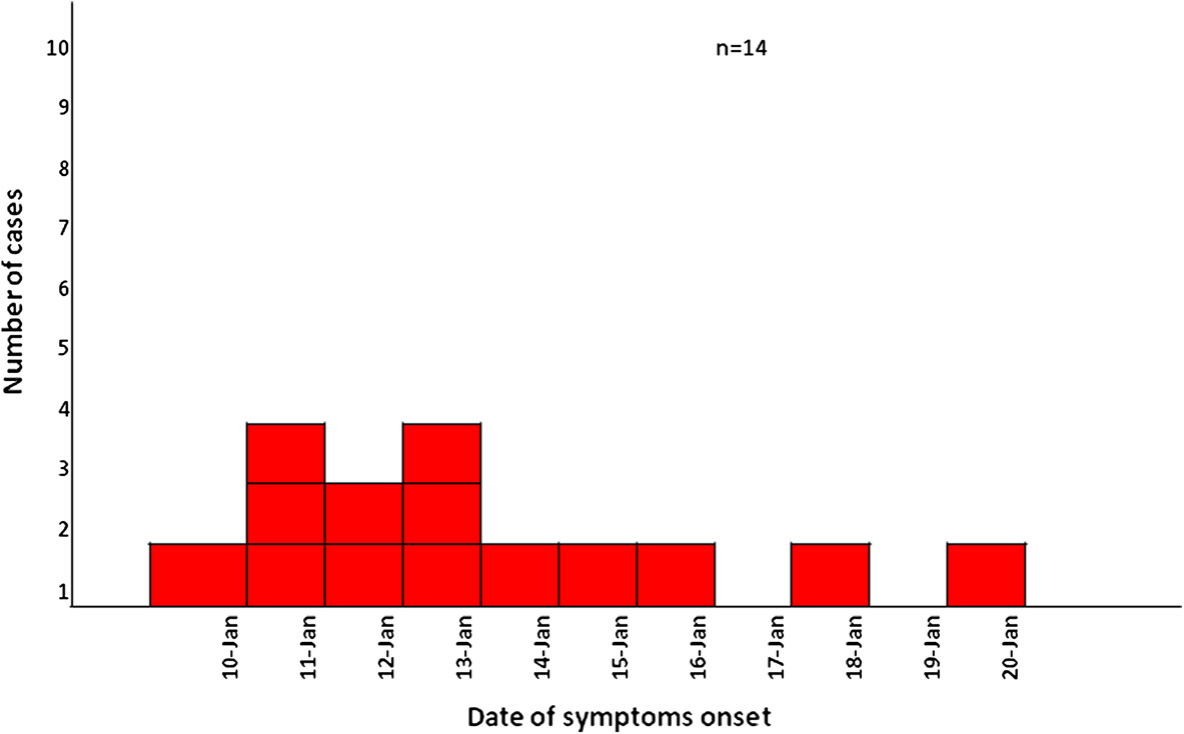
Date of illness onset for gastroenteritis cases (n = 14), Kindergarten, Nea Santa, Kilkis, January 2012.

The kindergarten population comprised of 45 people, 39 pupils, five teachers and one cleaning staff member, who was actually the same for both schools. Epidemiological information was collected through the structured questionnaire from 23 (response rate: 51.1%) of the kindergarten members. Information was acquired for 19 pupils (response rate: 48.7%), four teachers (response rate: 80.0%) and one staff member (100%). The median age of the 23 participants of the study was 5 years (min: 4, max: 45 years), with the vast majority of them (82.6%) being less than 6 years old; 13 (56.5%) of them were females. Of the 23 people, a total of 14 met the criteria of case definition (attack rate: 60.9%). Distribution of cases by date of symptoms onset is presented in Figure 2. The shape of the epidemic curve is compatible with person to person transmission of the disease. The first cases occurred one day after the cases at the elementary school. The cleaning staff member reported that her symptoms started on 18/01/2012. The attack rates among males and females were 60.0% and 61.5%, respectively. The majority of the reported cases (85.7%) were pupils; the attack rate for the kindergarten student population was 63.2%, while two teachers presented symptoms (attack rate 50.0%). The main reported symptoms of cases are presented in Table 1. The mean number of vomiting per day was 3 (min: 1, max: 10), while the mean number of diarrhoeas per day was 3 (min: 1, max: 7). Duration of illness ranged from 1 to 15 days, with a median of 2 days. Only one case (7.14%) was hospitalized. All 14 cases (100%) remained at home for recovery, while the median duration of absence from school was 5 days (2–15 days). Five cases (35.7%) reported a person in their environment with gastroenteritis symptoms before the onset of their symptoms, while 11 cases (78.6%) reported a symptomatic person in their environment after the onset of their symptoms.

Investigation did not reveal a specific relationship between children of the two schools (siblings or cousins). The univariate analysis revealed no statistically significant risk factor. Attack rates and relative risks are summarized in Table 3.

**Table 3:** Results of the univariate analysis, Kindergarten, Nea Santa, Kilkis, January 2012

	**Exposed**			**Non exposed**				
***Risk factors***	***Cases***	***Total***	AR*%	***Cases***	***Total***	AR*%	***RR****†*	***95%CI‡***
Consumption of tap water from the kindergarten	5	7	71.4	9	14	64.3	1.11	0.60-2.04
Consumption of bottled water	10	15	66.7	4	6	66.7	1.00	0.51-1.95
Another person at approximate environment with gastroenteritis symptoms before the onset of symptoms	5	5	100.0	9	9	100.0	1.00	1.00-1.00
Visit to playground	3	5	60.0	11	16	68.7	0.87	0.40-1.92
Participation in party	2	3	66.7	12	18	66.7	1.00	0.42-2.37

*Attack Rate *†Relative Risk ‡ 95% Confidence Interval*.

#### Environmental investigation

The schools are connected to the public water supply system and no problems had been recently reported and there had been no testing of the water the previous months. The temperature during the period before the occurrence of the outbreak was low (−3 to 9°C) and snowfall was heavy for several days. Hygiene inspection of the schools premises did not reveal any significant problem.

#### Laboratory investigation

The tap water sample from the kindergarten was positive for human AdV, while AdVs and NoVs were not detected in the sample from the elementary school. Water sampling and analysis performed by the municipal enterprise for water supply and sewerage of Kilkis showed that the measured standard bacterial parameters were within normal limits.

Eleven faecal samples were collected from seven pupils in total (two specimens from each of four pupils and one specimen from each of three pupils). All pupils were cases. Real-time (RT-) PCR results of fecal specimens’ nucleic acid samples tested positive for all selected viral targets (NoV GI, NoV GII, hAdV). Laboratory investigation showed that: a) two children were found positive for Norovirus GI, Norovirus GII and Adenovirus, b) one child was found positive for Adenovirus, c) one child was found positive for Norovirus GII.

## Discussion

We report on the investigation of two gastroenteritis outbreaks, in an elementary school and a neighboring kindergarten in a village with 1,538 residents in Northern Greece. Even though information from epidemiological and molecular investigation is of utmost importance to guide outbreak control measures and to prevent future outbreaks, gastroenteritis outbreaks at school settings are rarely investigated [19].

Evidence did not support the hypothesis of food-borne origin of the outbreak in the elementary school and cases could not be attributed to a single pupil or teacher. According to the results of the analytical epidemiological study, those who consumed tap water at school were more likely to develop gastroenteritis symptoms than those who did not consume tap water. The shape of the epidemic curve was compatible with a point source outbreak followed by secondary transmission. Even though the aetiological agent of the outbreak, was not verified, the epidemic curve in combination with the descriptive data, and the detection of NoV GI NoV GII, and AdVs in clinical samples collected from the elementary school pupils support the hypothesis of a mixed viral gastroenteritis outbreak. The detection of a viral pathogen/s was not possible at the tap water sample of the elementary school, but evidence of faecal contamination was found in one sample of tap water from the kindergarten, which was positive for hAdV; since the two schools have the same water supply system this finding is supportive of our hypothesis. Noroviruses (NoVs) and adenoviruses (AdVs) are common causative agents of gastroenteritis outbreaks in both children and adults and are believed to be implicated to several community outbreaks [20]. AdVs have been detected in waters of various origins and have been responsible for many waterborne outbreaks [21]. In addition, they have been proposed as indicators of human fecal contamination in water and food, since they are excreted by the populations of all geographical areas and are the most abundant viruses detected in urban sewage [22].

The fact that no Norovirus was detected in the analyzed water samples and that only the water sample from the kindergarten was found positive for AdVs can be explained by the fact that the two tap water samples were collected on 17 January, several days after the outbreak onset on 09 January and after the outbreak peak on 12/01/2012. NoVs and AdVs present different stability characteristics in the environment.

Financial constraints did not allow the molecular typing of the detected viral agents by sequencing.

## Conclusions

Verification of the aetiological agent of waterborne outbreaks is always challenging since most of them are of mixed etiology [23-25] and sampling is usually performed with delay [25,26]. In this outbreak, viruses could have already disappeared from the water or been present at much diluted concentrations in the supposed case of a transient contamination at the day of water sampling. Moreover, despite improvements in the methodology used, detection of viruses in water remains challenging and NoVs are not identified in water in most suspected Norovirus related waterborne outbreaks [27].

Based on the revised strength-of-evidence classification of waterborne outbreaks of the Centre for Diseases Control of USA [28], this outbreak could be considered as a class II waterborne outbreak based on the available epidemiological data. It is believed that heavy snowfall while the school was closed for 15 days and the fact that the water system of the schools was not used during that period contributed to an increased microbial load of the water that was consumed by teachers and pupils on the first day after holidays (9th January). Heavy rains and snowfalls contribute to run-off of water from the fields into the rivers through mobilization of pathogens in the environment [29-31]. The hypothesis that the water was contaminated with human or animal waste and that pathogens were multiplied during that period need further investigation.

Regarding gastroenteritis cases at the kindergarten, the shape of the epidemic curve refers to a person-to-person transmission and univariate analysis did not reveal a statistically significant risk factor. Diarrheic fecal samples were not collected, although recommended. The results of the investigation were not conclusive regarding the mode of transmission of the outbreak in the kindergarten. This was mainly because contacts were difficult to be defined due to the young age of children, many children were in diapers, and practices followed by the personnel inside the kindergarten could not be accurately documented. This viral gastroenteritis outbreak investigation is one of the very few that combined epidemiological, laboratory and environmental data in Greece; it highlights the value of molecular methods in outbreak investigations, and illustrates the importance of inter-agency collaboration.

Despite the limitations of this investigation, such as the delayed notification, the difficulty in collecting clinical and environmental samples early in the course of the outbreak, it clearly showed that the local public health authorities should play a more active role in the investigation of such outbreaks and that training of the personnel on the different aspects of outbreak investigation should be a future goal of HCDCP. Furthermore, since viral gastroenteritis outbreaks are frequent in school settings, school boards should be provided with a written protocol for timely notification of public health authorities and implementation of preventive measures. Recording of absenteeism at schools should be done on a daily basis and in a structured manner.

## Competing interest

The authors declare that they have no competing interests.

## Authors’ contribution

All authors (KM, TS, MP, PK, PZ, TG, AV) have been participated in the conception and design, acquisition of data or analysis and interpretation of data, the drafting of the article or revising it critically for important intellectual content as well as its final approval of the version published.

## Funding statement

This research received no specific grant from any funding agency in the public, commercial or not-for-profit sectors.

## License for publication

"The Corresponding Author (Dr Apostolos Vantarakis) has the right to grant on behalf of all authors and does grant on behalf of all authors, an exclusive license (or non exclusive for government employees) on a worldwide basis to the BMJ Publishing Group Ltd and its Licensees to permit this article (if accepted) to be published in JECH editions and any other BMJPGL products to exploit all subsidiary rights, as set out in our license.

## Pre-publication history

The pre-publication history for this paper can be accessed here:

http://www.biomedcentral.com/1471-2458/13/241/prepub
